# New trend in artificial intelligence-based assistive technology for thoracic imaging

**DOI:** 10.1007/s11547-023-01691-w

**Published:** 2023-08-28

**Authors:** Masahiro Yanagawa, Rintaro Ito, Taiki Nozaki, Tomoyuki Fujioka, Akira Yamada, Shohei Fujita, Koji Kamagata, Yasutaka Fushimi, Takahiro Tsuboyama, Yusuke Matsui, Fuminari Tatsugami, Mariko Kawamura, Daiju Ueda, Noriyuki Fujima, Takeshi Nakaura, Kenji Hirata, Shinji Naganawa

**Affiliations:** 1 Department of Radiology, Osaka University Graduate School of Medicine, 2-2 Yamadaoka, Suita-City, Osaka 565-0871 Japan; 2 Department of Radiology, Nagoya University Graduate School of Medicine, 65 Tsurumai-cho, Showa-ku, Nagoya, Aichi 466-8550 Japan; 3 Department of Radiology, Keio University School of Medicine, 35 Shinanomachi, Shinjuku-ku, Tokyo, 160-0016 Japan; 4 Department of Diagnostic Radiology, Tokyo Medical and Dental University, 1-5-45 Yushima, Bunkyo-ku, Tokyo, 113-8519 Japan; 5 Department of Radiology, Shinshu University School of Medicine, 3-1-1 Asahi, Matsumoto, Nagano 390-2621 Japan; 6 Department of Radiology, Graduate School of Medicine and Faculty of Medicine, The University of Tokyo, 7-3-1 Hongo, Bunkyo-ku, Tokyo, 113-8655 Japan; 7 Department of Radiology, Juntendo University Graduate School of Medicine, Bunkyo-ku, Tokyo, 113-8421 Japan; 8 Department of Diagnostic Imaging and Nuclear Medicine, Kyoto University Graduate School of Medicine, 54 Shogoin Kawaharacho, Sakyoku, Kyoto, 606-8507 Japan; 9 Department of Radiology, Faculty of Medicine, Dentistry and Pharmaceutical Sciences, Okayama University, 2-5-1 Shikata-cho, Kita-ku, Okayama, 700-8558 Japan; 10 Department of Diagnostic Radiology, Hiroshima University, 1-2-3 Kasumi, Minami-ku, Hiroshima, 734-8551 Japan; 11 Department of Diagnostic and Interventional Radiology, Graduate School of Medicine, Osaka Metropolitan University, 1-4-3 Asahi-Machi, Abeno-ku, Osaka, 545-8585 Japan; 12 Department of Diagnostic and Interventional Radiology, Hokkaido University Hospital, N15, W5, Kita-ku, Sapporo, 060-8638 Japan; 13 Department of Diagnostic Radiology, Kumamoto University Graduate School of Medicine, 1-1-1 Honjo Chuo-ku, Kumamoto, 860-8556 Japan; 14 Department of Diagnostic Imaging, Graduate School of Medicine, Hokkaido University, Kita 15 Nish I 7, Kita-ku, Sapporo, Hokkaido 060-8648 Japan

**Keywords:** Artificial intelligence, Deep learning, Convolutional neural network, Vision transformer, Explainable AI, Thoracic imaging

## Abstract

Although there is no solid agreement for artificial intelligence (AI), it refers to a computer system with intelligence similar to that of humans. Deep learning appeared in 2006, and more than 10 years have passed since the third AI boom was triggered by improvements in computing power, algorithm development, and the use of big data. In recent years, the application and development of AI technology in the medical field have intensified internationally. There is no doubt that AI will be used in clinical practice to assist in diagnostic imaging in the future. In qualitative diagnosis, it is desirable to develop an explainable AI that at least represents the basis of the diagnostic process. However, it must be kept in mind that AI is a physician-assistant system, and the final decision should be made by the physician while understanding the limitations of AI. The aim of this article is to review the application of AI technology in diagnostic imaging from PubMed database while particularly focusing on diagnostic imaging in thorax such as lesion detection and qualitative diagnosis in order to help radiologists and clinicians to become more familiar with AI in thorax.

## Introduction

Looking back on the history of artificial intelligence (AI) [1–3], the first AI boom emerged in the 1950s, when computational reasoning and exploration flourished and expectations for AI grew. The next boom was the second AI boom in the 1980s. A system that responds with conditioned reflexes by teaching AI knowledge in the form of rules, called an expert system, was put into practical use. Machine learning is a technique in which a program learns from data, extracts patterns, and makes predictions and judgments on unknown data. Typical algorithms include support vector machines, random forests, and decision trees. Deep learning is a branch of machine learning based on hierarchical and multilayer artificial neural networks. Deep learning appeared in 2006, and it has been more than 10 years since the third AI boom was triggered by improvements in computing power, algorithm development, and the use of big data. Recent advancements in AI, particularly in deep learning, have revolutionized the field of medical image analysis. In the past, medical image analysis relied heavily on systems created by human domain experts, utilizing statistical or machine learning models that required manual selection of image features or regions of interest [4]. However, recent deep learning models can automatically learn these image features with minimal human intervention, resulting in more efficient and resource-saving image analysis tasks [5]. Currently, AI techniques have been developed for various fields of medical imaging including chest radiography, computed tomography (CT), magnetic resonance imaging (MRI), positron emission tomography (PET-CT), and ultrasonography [6–23]. In the thorax, particularly, AI has already been applied in clinical settings, such as nodule detection support, benign/malignant diagnosis support, image processing such as automatic quantification, and image quality improvement through noise reduction processing.

This article mainly outlines detection, diagnosis, and prognosis, focusing on image recognition, lung cancer as a representative lung tumor, coronavirus disease 2019 (COVID-19) as an infectious disease, and pulmonary embolism (PE) as a non-tumor/non-infection. This review can help readers understand the recent trends in AI in the field of thoracic imaging area and how to use AI as it evolves.

## Materials and methods

**Fig. 1:**
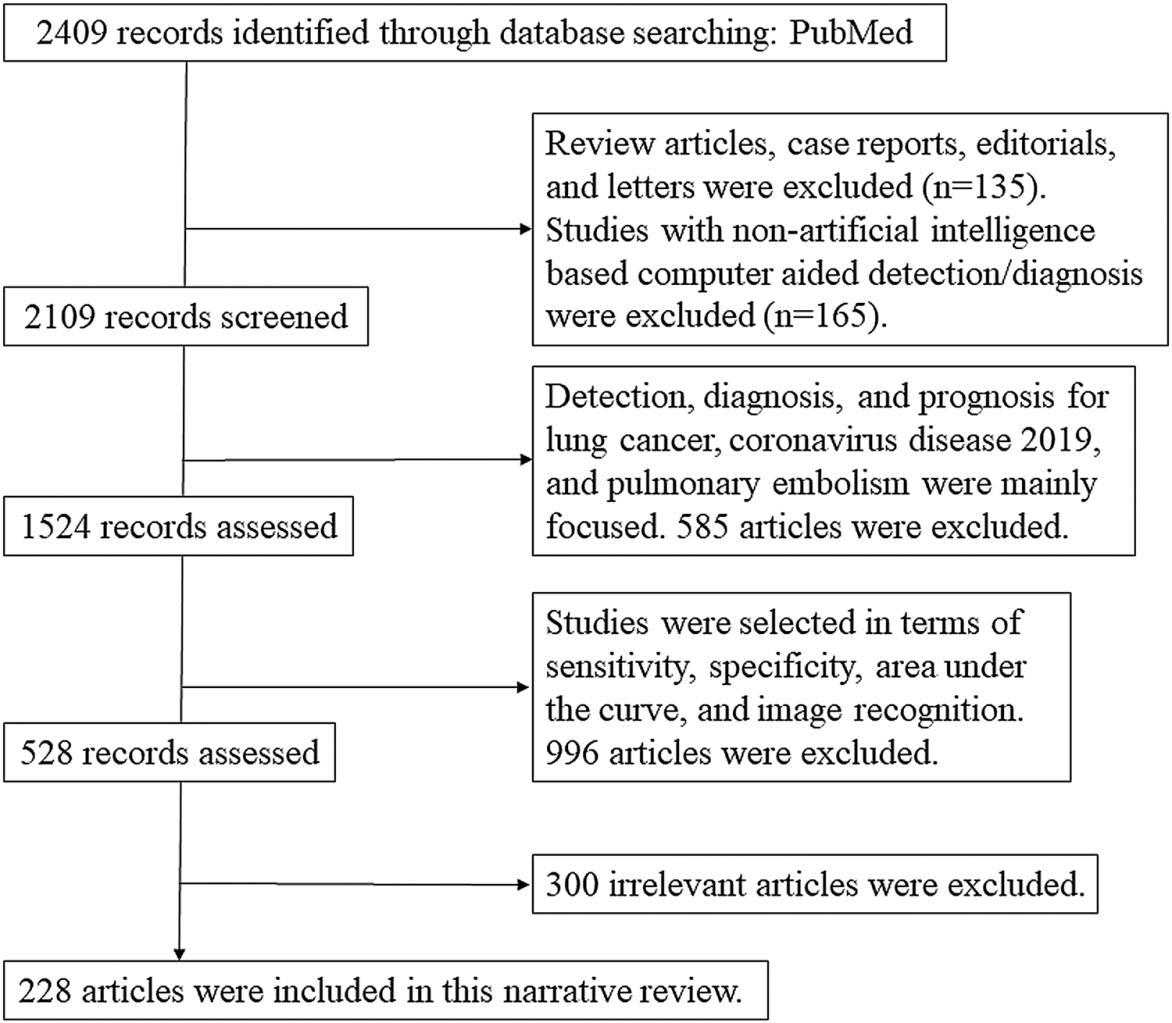
Flowchart of studies selection process

For the literature survey, PubMed database up to April 30, 2023, was used to search studies relevant to evaluation of clinical applications in thorax using AI. Keywords searched included the following: {lung imaging “English” [Language] AND (“artificial intelligence” OR “deep learning” OR “neural network” OR “machine learning” OR “computer aided”) AND (“CT”) NOT (“ultrasonography” OR “PET” OR “SPECT”)}. In particular, detection, diagnosis, and prognosis for lung cancer, COVID-19, and PE were mainly focused in this review. Citations and references from the retrieved studies were used as additional sources for literature review, and manually search was conducted. Review articles, case reports, editorials, and letters were excluded. Exclusion criteria were as follows: (a) researches outside the subject area; (b) phantom or animal or healthy individual researches; (c) researches focused on methodological aspects alone; (d) researches by a variety of methods; (e) researches on organs other than the lung; and (f) researches without evaluation of clinical outcomes. Figure 1 shows the flowchart of the selection process. Finally, 228 articles were included in the narrative review.

### AI mechanisms commonly used for image recognition

The model commonly used in image recognition is a convolutional neural network (CNN), in which the convolution and pooling layers are repeated to produce the output, while performing image pattern matching, data compression, and misalignment correction. The CNNs have been used in many AI researches [6–22], which have shown promising performance in detecting and diagnosing diseases on medical imaging, sometimes outperforming radiologists [7, 19, 24]. However, one of the problems of AI is overfitting [25]. That is, AI works for known training data but does not fit for unknown data due to limitations caused by the generalizability of the variables under the shift of the data distribution. In addition, the high degree of freedom in the design of hidden layers is a factor in the excellent expressiveness of neural networks, but hidden stratification may cause better or worse performance of CNN models on certain subsets [26, 27]. CNN models provide excellent output but leave room for improvement.

As a relatively new image recognition model, a model called vision transformer (ViT), which does not use the convolution operation, has appeared [28, 29] and has shown promising performance on general image datasets. The ViT model processes images not as pixels but as patches of a certain size by applying techniques used in natural language processing to image processing, which uses an analogous approach by dividing an input image into some pixel patches and projecting them into a linear embedding. The sequence is input to the transformer encoder, which contains multi-headed self-attention layers and multilayer perceptron blocks after layer normalization to reduce training time and improve generalization performance. The transformer encoder outputs feature vectors corresponding to the input patches, followed by softmax activation. Due to the parallel processing by multi-headed self-attention layers and multilayer perceptron blocks, there is an advantage in terms of computational cost, and there is also an advantage that performance does not degrade even when the model is large. Recently, Murphy et al. [30] compared that ViT and CNN for disease classification on chest radiographs. CNN models consistently outperformed ViT models, although the absolute differences in performance were small and may not be clinically significant. On the other hand, ViT was less susceptible to specific kinds of hidden stratification known to affect CNN models. At this time, it may be too early for ViT to be a replacement for CNN in the diagnosis of disease on chest radiographs. Although ViTs have shown improvements over CNNs in general imaging datasets [29, 31], the usefulness of ViT must be further examined in the future.

### Clinical application to lung tumors: detectability of pulmonary nodules

The leading cause of cancer-related deaths is lung cancer [32], and in our country, the mainstream approach to reducing lung cancer mortality is screening with chest radiography. The results of two randomized controlled trials, the National Lung Screening Trial (NLST) in the USA in 2011 [33] and the Dutch-Belgian randomized lung cancer screening trial (NELSON) in Europe in 2020 [34], suggest that low-dose CT screening may be effective in reducing lung cancer mortality rates among heavy smokers. Therefore, the need for low-dose CT screening should be considered internationally. In screening and routine clinical practice, the detection of numerous pulmonary nodules on chest radiography or chest CT is an important task for physicians. However, increased workload may lead to physician burnout [35].

**Fig. 2:**
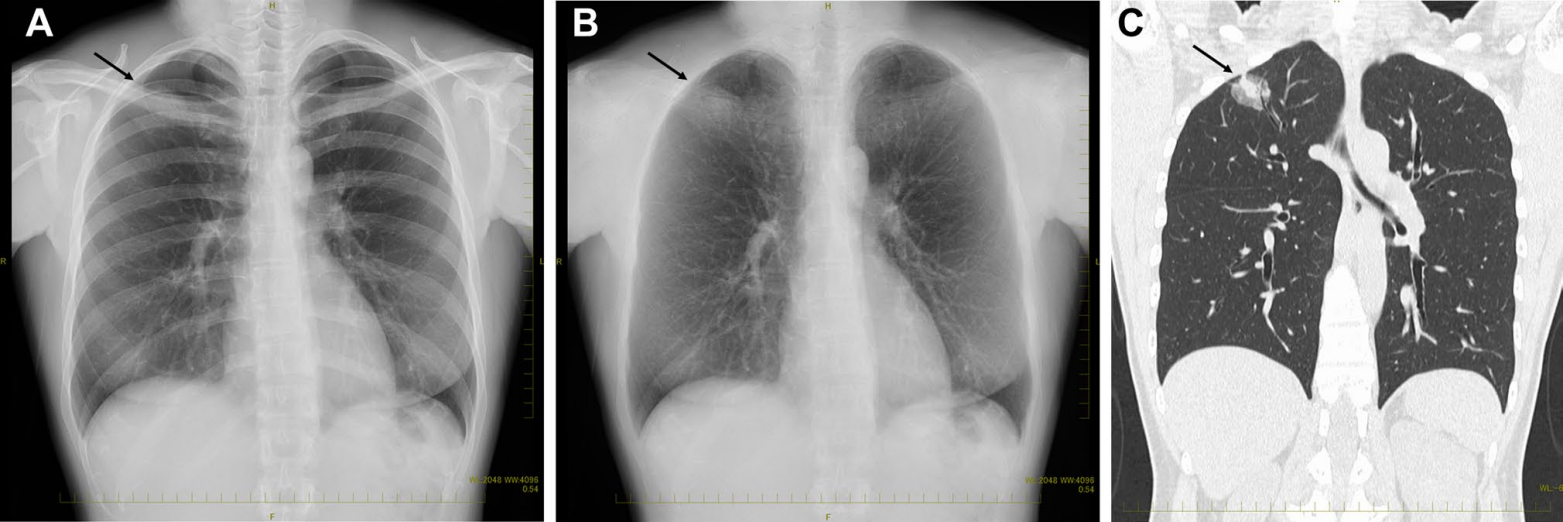
Nodule detection on chest radiograph with bone suppression by a commercially available AI model. A 42-year-old woman. A low radiolucent area is suspected on the right supraclavicular bone (arrow, **A**). However, it is difficult to distinguish between pulmonary and bone lesions. A pulmonary nodule hidden behind the right clavicle can be detected on the chest radiograph with bone suppression using a commercially available AI model (arrow, **B**). A part-solid ground-glass nodule with about 3 cm in diameter can be seen in the right upper lobe (arrow, **C**). This nodule was confirmed as a lung adenocarcinoma after surgery. *AI* artificial intelligence

Recent studies have shown that AI has high performance in detecting pulmonary nodules on chest radiographs and CT scans [36–41]. In the detection studies reviewed for chest radiography using the AI-based computer-aided detection/diagnosis (CAD) system [37, 39, 40], the sensitivity ranges from 79.0 to 91.1% and the specificity from 93 to 100%. The value of the false positive (FP) per scan is between 0.02 and 0.34. Moreover, the AI-based CAD system with bone suppression technology was more sensitive to detecting pulmonary nodules on chest radiographs compared with the original model by improving the visibility of pulmonary nodules hidden behind the clavicle and ribs (Fig. 2) [42]. On the other hand, in the detection studies reviewed for CT using the AI-based CAD system [37, 38, 43], the sensitivity ranges from 61.6 to 98.1%, and the value of the FP per scan ranges from 0.125 to 32. Pulmonary nodules have been already detected with high area under the curve (AUC) values close to 1. However, studies have reported a wide range of FP rates for AI detection of pulmonary nodules in chest radiographs and CT scans, as shown above [36–40]. The variability in reported FP rates may be due to differences in the AI models used, the populations studied, and the specific evaluation metrics used. It is important to note that reducing FP rates is a key challenge for improving the accuracy and effectiveness of AI-based nodule detection systems.

**Fig. 3:**
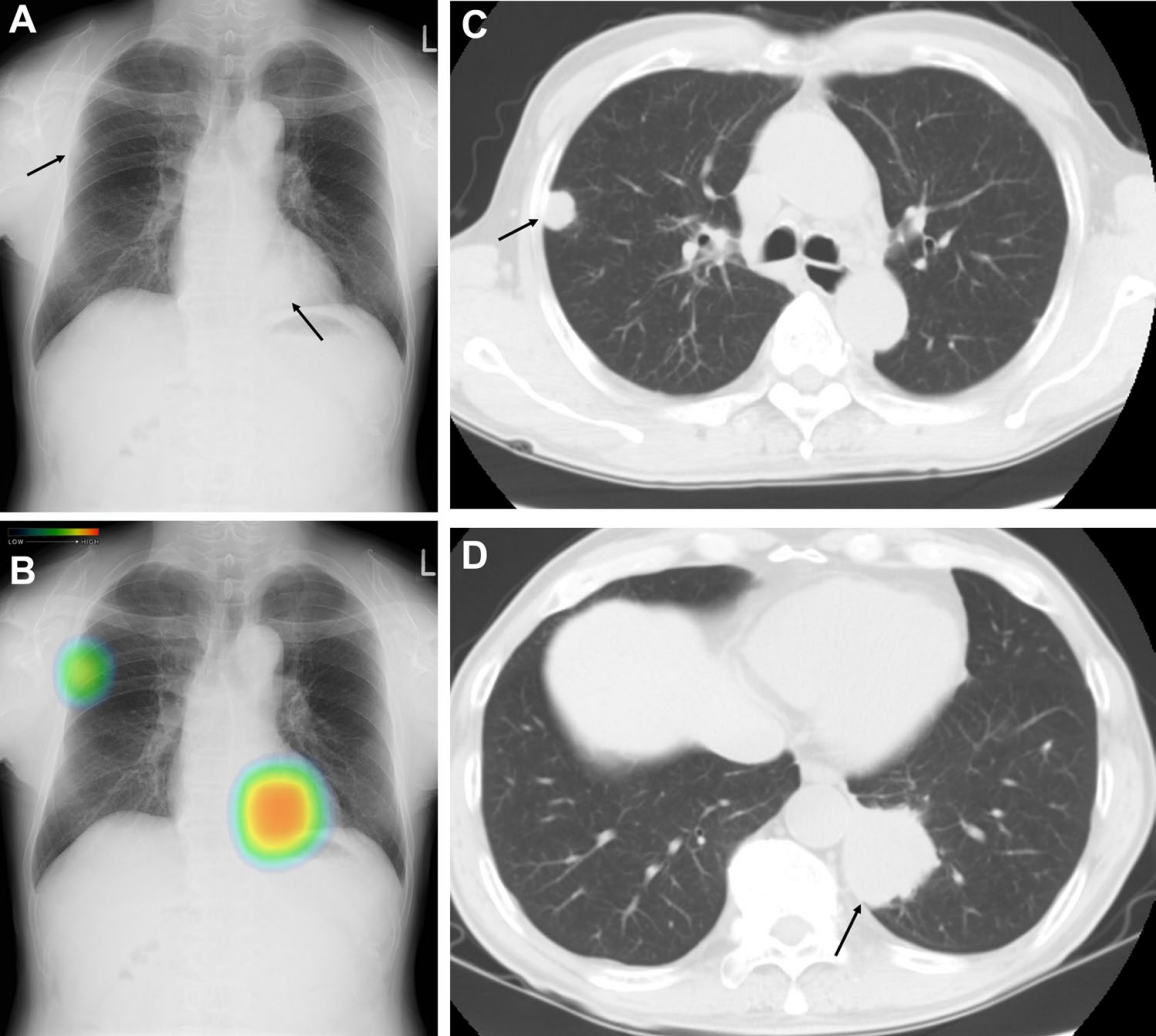
Nodule detection on chest radiograph by a commercially available AI-based CAD system. A 70-year-old man. A nodule can be seen in the right middle lung field and a mass can be seen in the left lower lung field overlapping the cardiac shadow (arrows, **A**). A commercially available AI-based CAD system can highlight both the nodule and mass. A nodule with 2 cm in diameter can be seen in the right upper lobe on CT (arrow, **C**). A mass with 5 cm in diameter can be seen in the left lower lobe on CT (arrow, **D**). This mass was confirmed as a lung metastasis from renal cell carcinoma after biopsy. *AI* artificial intelligence, *CAD* computer-aided detection/diagnosis

AI-based CAD system has already outperformed physicians in nodule detection performance on chest radiography and CT, and it enhanced physicians’ performances when used as a second reader [40, 44]. The AI-based CAD system is useful in medical examinations and daily clinical practice to prevent physicians from overlooking pulmonary nodules and is also useful as a diagnostic support in areas with a shortage of physicians (Fig. 3). However, there are still many challenges and limitations remaining including overfitting, lack of interpretability, and insufficient annotated data. A generative model via an adversarial setting called generative adversarial network (GAN), proposed by Goodfellow et al. [45], has been widely used to generate synthetic images for medical model development. Madani et al. [46] demonstrated that semi-supervised GANs can achieve superior performance compared to conventional supervised CNNs, while using less training data. Although GANs are less prone to overfitting, their training process can be unstable due to the Nash equilibrium between the generator and the discriminator. The use of GANs in the medical domain may raise ethical concerns. In order to give AI the same sense of ethics as humans, it is crucial to improve the process by reviewing biases in the knowledge and information used for training in advance. Care must be taken to ensure that seemingly positive results do not lead to biased outcomes and judgments. Further studies are needed to investigate the generalizability and clinical utility of an AI-based CAD system on chest radiography and CT.

### Clinical application to lung tumors: diagnosability of pulmonary nodules

Detection of lung cancer in its early nodular stage through screening, categorization, and medical care has proven to be highly beneficial and effective in reducing mortality rates associated with lung cancer. Qualitative and quantitative evaluations using images play an important role in the diagnosis of thoracic tumors including lung cancer [47–61]. Diagnosis of the malignancy or benignity of pulmonary nodules is also one of the critical tasks for physicians, but it is commonly acknowledged that achieving 100% accuracy in determining the malignancy or benignity of nodules based on CT images alone is unattainable. As a result, in many cases, the diagnosis of CT images without histopathologic diagnosis requires reliance on follow-up observations. Therefore, it would be reassuring for physicians to have AI support for qualitative diagnosis. For the early detection and diagnosis of lung cancer, various AI-based CAD systems have been developed to classify benign and malignant lung nodules [62–65]: AI-based CAD system with 2-dimensional (2-D) or 3-D CNN network achieved good classification performance in malignant suspicion of pulmonary nodules with close to 90% classification accuracy. In past reports [62–65], most of the existing CNN-based approaches use a 2D-CNN network due to the advantages of low network complexity and fast computation. However, compared with the 2D-CNN network, the 3D-CNN network can encode richer spatial information and extract more representative features through its hierarchical architecture trained with 3D samples, which also contributes to the efficient detection of nodules to be qualitatively diagnosed by reducing the FP rates in automated pulmonary nodule detection on CT [66], while requiring a large amount of data and taking a long time to compute. Parallel processing of ViT models [28, 29] is expected to improve diagnostic performance in the future because it has an advantage in terms of computational cost and its performance does not deteriorate easily even if the model size is increased, but the need to prepare huge data for model training is the same as for CNN models. The potential benefits of ViT in improving diagnostic accuracy should be further investigated in the future.

As with nodule detection, an AI-based CAD system can have at least some impact on physician diagnosis. An AI model predicting the pathological invasiveness of lung adenocarcinoma, constructed from a small dataset of 285 cases, showed diagnostic performance with an accuracy of 74.2%, a sensitivity of 80.3%, a specificity of 67.1% [7], and an AUC value of 0.74. When this model was used by three different physicians with 9, 14, and 26 years of experience, sensitivity was improved at the expense of specificity. There was no significant difference in overall diagnostic performance, but the accuracy of the less experienced physician was significantly improved. Although it is difficult for physicians to predict the invasiveness of lung adenocarcinoma manifested as ground-glass nodules on CT images, the combination of physicians with AI-based CAD system using CNN network can outperform physicians alone in internal dataset (AUC values, 0.819 vs. 0.606) and external dataset (AUC values, 0.893 vs. 0.693) [67]. In addition, CNN may be beneficial in improving the accuracy of computerized detection of volume measurements and the ability to differentiate nodules on CT scans in patients with pulmonary nodules [68]. Wataya et al. [69] demonstrated that a commercially available AI-based CAD system could measure the accuracy of imaging features of nodules/masses with high interobserver agreement, thereby improving the accuracy of benign or malignant diagnosis. In particular, the AI-based CAD system was valuable for radiologists with less experience. AI-based CAD system for diagnosis will be very promising in the future.

### Clinical application to lung tumors: prediction of prognosis

Many approaches have been investigated to develop deep learning prognostic models for lung cancer patients using CT scans. In particular, the ability to predict preoperative outcomes using CT data would be extremely beneficial for individuals diagnosed with early-stage lung cancer, as it would assist in determining appropriate treatment strategies [70–72].

**Fig. 4:**
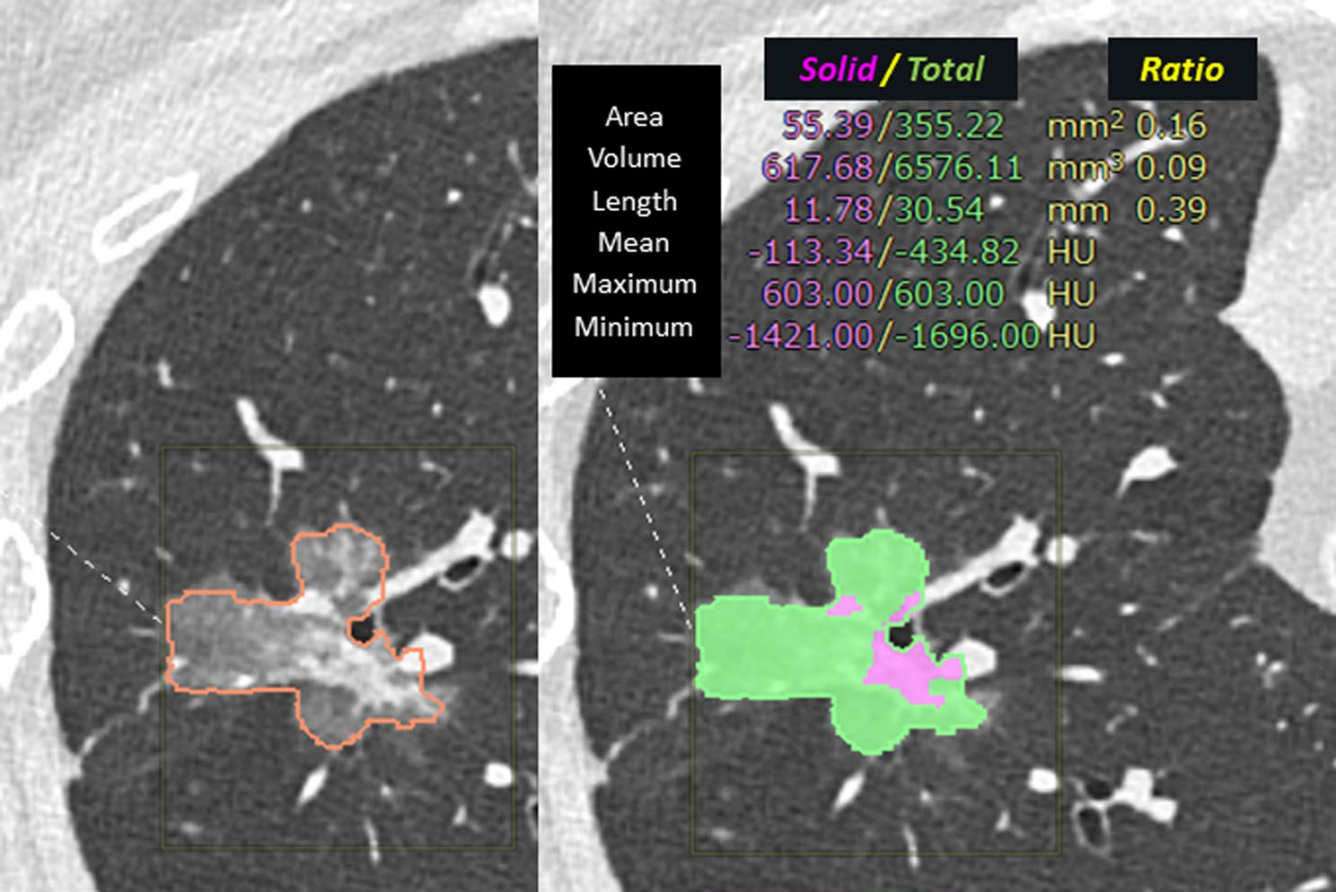
Quantification by a commercially available AI-based CAD system. A commercially available AI-based CAD system can automatically segment a part-solid ground-glass nodule, providing quantitative values that are exactly the same regardless of the observers. *AI* artificial intelligence, CAD computer-aided detection/diagnosis

In the clinical setting, measuring the size of invasiveness on CT for the T descriptor size is very important in the 8th edition of the TNM lung cancer classification because the T descriptor size correlates with the patients’ prognosis [73, 74]. To decide the T descriptor of lung cancer, the solid component on CT is measured in centimeters (to one decimal place) with a slice thickness of 1 mm or less in the lung field conditions (recommended). Measurements are usually assessed in cross section, but the lack of standardized methods for tumor characterization and measurement remains a problem. Therefore, measurements are dependent on viewing conditions and observers, resulting in variability. Quantitative analysis using 3D volumetry is more advantageous in terms of objectivity and reproducibility of measurements [75, 76]. Wormanns et al. [76] demonstrated that a 25% increase in measured volume had a 95% probability of reflecting true nodule growth rather than measurement variability. However, differences in conditions such as software performance and nodule characteristics cause some errors in measurements, whether between the same observer or between different observers. Even if the software is the same, care must be taken when comparing measurements. On the other hand, a recent commercially available AI-based CAD system [69], which is already in clinical use in our country, can automatically provide quantitative values and exactly the same values regardless of display conditions and observers (Fig. 4). This AI-based CAD system can provide quantitative values with extremely high accuracy and reproducibility. Volumetric measurements, such as solid volume and solid volume percentage, have been found to be independent indicators associated with an increased likelihood of recurrence and/or death in lung cancer patients [77]. Highly accurate quantification using an AI-based CAD system may lead to highly reproducible prognostic prediction in clinical practice.

A recent study put forth a deep learning model that directly predicted the cumulative survival probability in patients diagnosed with early stage lung adenocarcinoma using preoperative CT data [72]. Multivariate analysis data demonstrated smoking status (hazard ratio [HR], 3.4; *p* = 0.007) and the output of the deep learning model (categorical form: HR, 3.6; *p* = 0.003) were the only independent prognostic factors for disease-free survival in patients with clinical stage I lung adenocarcinoma. However, this approach had some limitations in terms such as the time-to-event data used to train the model and the presence of censoring, particularly in early-stage lung cancer patients. Prediction of prognosis directly from images may output highly reproducible results, but due to the nature of AI, whose analysis process is a black box, it may sometimes be difficult to accept the final results of AI. Zhong et al. [78] reported a prognostic stratification AI of lymph node metastasis using based on the AI score calculated from the CT image data of the primary lung cancer, which may be more acceptable to us. In the future, we hope to build a more accurate prognosis prediction model by learning clinical data such as many morphological evaluations and histological characteristics. In addition, so-called ablation studies, in which the elements that make up the model are deliberately deleted to evaluate how these elements contribute to the performance of the model, are also considered important in the construction of the model.

### Clinical application for infectious diseases: focusing on COVID-19

Although there are many respiratory infections, many infectious disease AIs naturally target COVID-19, formally known as severe acute respiratory syndrome coronavirus 2 [79, 80]. In general, chest radiography and CT are indispensable for diagnostic imaging of chest diseases including infectious diseases [81, 82]. The usefulness of chest radiography and CT for COVID-19 associated with diagnosis, disease severity, and prognosis has been reported [83–90]. Radiologists exhibit a remarkably high proficiency in identifying COVID-19 pneumonia through the assessment of chest CT scans [91]. To combat the rapid spread of COVID-19, many AI models using imaging findings have been developed worldwide [12, 80]. Ito et al. demonstrated that evaluating the use of AI in diagnostic imaging for COVID-19 patients has the potential to serve as a pilot study for determining the ideal AI systems to be employed during the initial phases of future emerging diseases, providing valuable insights into the requirements for dataset size and the appropriate application of AI in such scenarios [80].

As an initial representative paper using chest CT, Li et al. [92] created an AI program using CT scans to identify individuals with COVID-19 pneumonia. The program demonstrated a sensitivity of 90%, a specificity of 96%, and an AUC of 0.96 for diagnosing COVID-19 pneumonia. A dataset of 400 COVID-19 patients, 1396 patients with community-acquired pneumonia, and 1173 patients with normal CT scans or without pneumonia was used to train the program. The performance of the program was assessed using a separate dataset consisting of 68 COVID-19 patients, 155 patients with community-acquired pneumonia, and 130 patients with normal CT scans or without pneumonia. The COVID-19 cases in the dataset were confirmed through reverse transcription polymerase chain reaction test. Although the specific details of the dataset were not disclosed, the source code was made available and could be visualized using gradient-weighted class activation mapping (Grad-CAM). The study encompassed a large dataset and achieved a high level of accuracy. However, various AI models for COVID-19 pneumonia classification using chest radiography and chest CT that have appeared since the early days of the COVID-19 have been in a mixed state, with conspicuous variations in databases and analysis methods [80, 93]. Early-stage AI results may have been more influenced by morbidity than by imaging findings. Hence, both sensitivity and specificity ranged from high to low compared to other AI creation researches, which seems to indicate the difficulty of actual AI operation. On the other hand, recent AI models of COVID-19 pneumonia have improved the accuracy of diagnosis and severity prediction through the use of highly accurate lesion segmentation techniques, quantitative methods including radiomics approaches, and combination with clinical information. The meta-analysis [94] revealed that AI models based on chest imaging discriminate COVID-19 from other pneumonias: pooled AUC of 0.96 (95% confidence interval [CI] 0.94–0.98), pooled sensitivity of 0.92 (95% CI 0.88–0.94), pooled specificity of 0.91 (95% CI 0.87–0.93). As in other areas, future researchers should pay more attention to the quality of research methodology and further improve the generalizability of the developed predictive models, while considering the importance of database construction, uniformity of evaluation methods, and the necessity of benchmarking the evaluation database.

### Clinical application for non-tumor/non-infection: focusing on pulmonary embolism

This section focused on PE, although there are many reports on the usefulness of thoracic imaging in non-oncologic and non-infectious diseases [95–97]. PE carries a high burden of morbidity and mortality. There is also a report demonstrated that the detection of an incidental PE on a staging CT scan is associated with a very high risk of progressive malignancy [98]. Timely and precise diagnosis enables the acceleration of treatment, a crucial factor in potentially reducing mortality rates and enhancing patient outcomes [99–101]. CT pulmonary angiography (CTPA) is the diagnostic standard for noninvasive confirmation of PE. Either dual-energy or single-energy CT can be used comparably for the detection of acute PE [102, 103]. Nevertheless, the identification of PE through CTPA is a tedious procedure that necessitates the expertise of radiologists. Consequently, the interpretation process is prone to error and may lead to delayed diagnoses.

An innovative approach to PE detection has been developed using AI, which includes three main tasks: classification to label an entire image, detection to localize an individual object in the image, and segmentation to delineate a pixel-wise border of emboli. Meta-analysis results [104] demonstrated that AI-based CAD system for PE indicated a pooled sensitivity of 0.88 (95% CI 0.803–0.927) per scan and a specificity of 0.86 (95% CI 0.756–0.924) per scan, which showed a better balance between sensitivity and specificity compared to radiologists’ sensitivity of 0.67–0.87 with a specificity of 0.89–0.99. The effective AI models will support physicians by reducing the rate of missed findings, thereby helping to minimize the time required to review the CT scans. Cheikh et al. [105] demonstrated that AI for the detection of PE seemed to serve as a safety net rather than a replacement for radiologists in emergency radiology practice. This is attributed to its high sensitivity and negative predictive value (NPV), which subsequently increases the confidence of radiologists. Batra et al. [106] retrospectively evaluated the performance of an AI algorithm in detecting incidental PE on enhanced chest CT examinations in 2555 patients. The AI algorithm was applied to the images, and a natural language processing (NLP) algorithm was used to analyze the clinical reports. A multi-reader adjudication process was implemented to establish a reference standard for incidental PE. The AI detected 4 incidental PEs missed by clinical reports, while the clinical reports identified 7 incidental PEs missed by the AI. The AI exhibited lower positive predictive value (PPV) and specificity than clinical reports, but there were no significant differences in sensitivity and NPV. The AI showed high NPV and moderate PPV in detecting incidental PE, complementing the radiologists’ findings by identifying some cases missed by them. Potential applications of the AI model serve as a second reader to help allow earlier PE detection and intervention, and improving the model by understanding AI misclassifications.

Patients with COVID-19 pneumonia are susceptible to developing deep vein thrombosis and PE. Prophylactic use of low-molecular-weight heparin does not reduce the risk of venous thromboembolism in COVID-19 pneumonia [107, 108]. Therefore, the performance of a commercially available AI algorithm for PE detection on enhanced CT in patients hospitalized for COVID-19 was investigated [109]. The AI algorithm demonstrated high sensitivity of 93.2% (95% CI 0.906–0.952) and specificity of 99.6% (95% CI 0.989–0.999) for PE on enhanced CT scans in patients with COVID-19 regardless of parenchymal disease. However, accuracy was significantly affected by the mean attenuation of the pulmonary vasculature, which may warrant further investigation in the future.

## Notes and expectations for future AI

The use of AI techniques in thoracic CT imaging has been shown to be beneficial for a number of fronts such as detection, diagnosis, and prognosis. AI can provide this information in a more objective, reproducible, and robust manner compared to subjective analysis and conventional quantification. Overfitting as one of the problems of AI, which is learned on the training data but not adapted to the unknown data, still remains. Building AI models by training on a large amount of data is ideal, but it is difficult to create a perfect one. Unsupervised learning is used when labeled data is limited or when the goal is to understand the features and structures of the data or to gain new insights. However, compared to supervised learning, unsupervised learning poses challenges in evaluating predictive performance and optimization. On the other hand, simply adding more training data does not always seem appropriate. CNNs trained on a modest collection of prospectively labeled data might achieve high diagnostic performance in classifying their data as normal or abnormal [110]. Care should also be taken when enriching data to improve generalizability. Methodological pitfalls have the potential to produce a superficially well-performed AI model because oversampling or augmenting data before it is divided into training, test, and validation data violates data independence [111].

Another AI problem is the black-boxing of the analysis process.

It is possible to identify features within the image on which DL focuses by using Grad-CAMs, attention map, and local interpretable model-agnostic explanations (LIME).

**Fig. 5:**
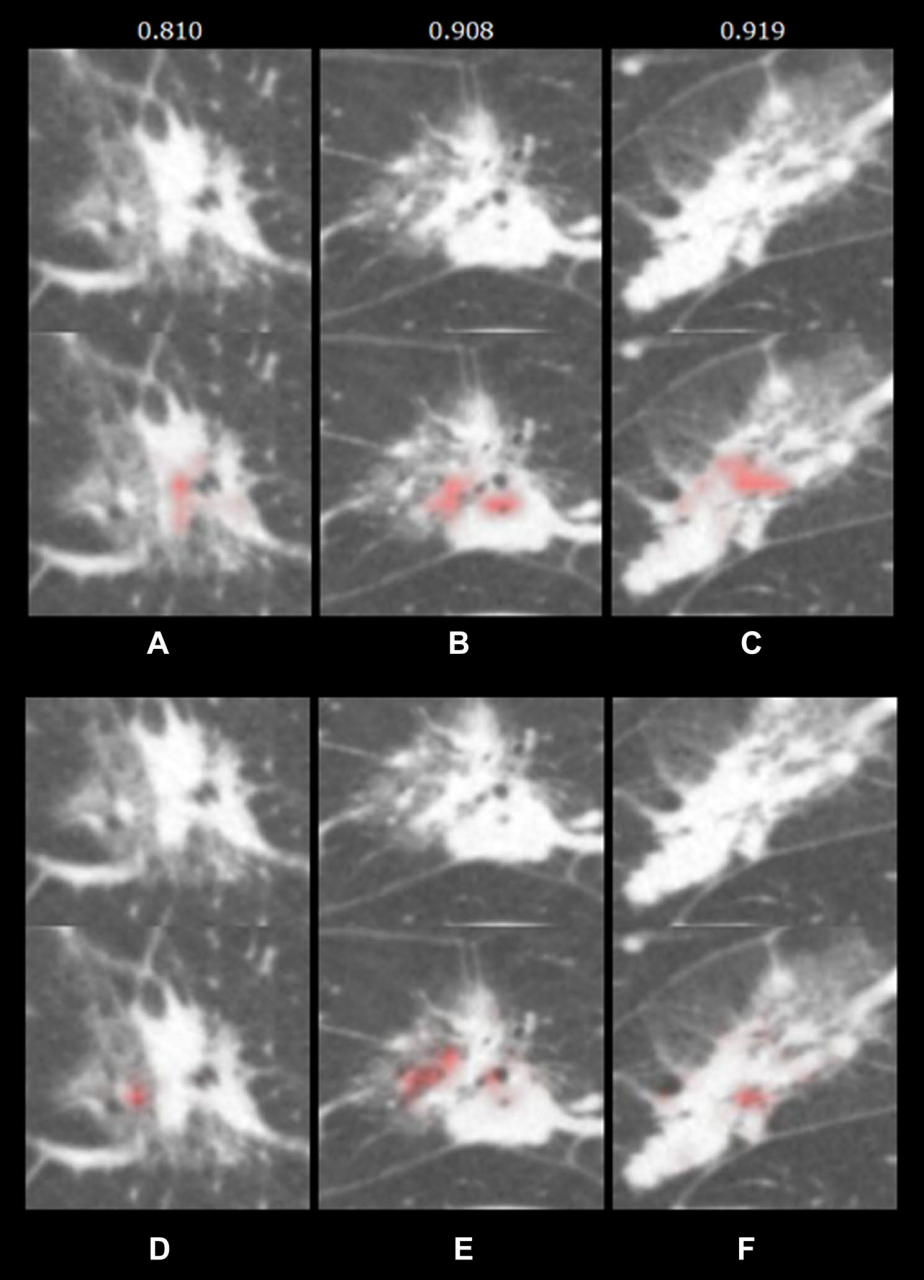
Differences of attention map in the diagnosis of lung nodule. This is a case confirmed as invasive adenocarcinoma after surgery (**a** and **d**, axial images; **b** and **e**, coronal images; **c** and **f**, sagittal images). Red areas in **a**, **b**, and **c** are focused by the AI in the diagnosis of invasive adenocarcinoma. Even for the same nodule, red areas in **d**, **e**, and **f** are focused when diagnosed as noninvasive adenocarcinoma. The numbers in the upper row indicate the diagnostic probability of invasive adenocarcinoma (maximum value of 1) calculated by the AI. Depending on the purpose of diagnosis and the cross section of the image, the focused part and the probability of results by the AI may differ. *AI* artificial intelligence

These heat maps cannot tell us what an AI concretely analyzed, but at least tell us where it focused (Fig. 5), which might prevent the possibility of unexpected findings being used to make a final decision due to the black box in the process of an AI. Further quantitative analysis of the focused lesions and correlation with pathology may be useful in constructing an explainable AI. It is desirable to build an end-to-end explainable AI that integrates various functions.

Imaging in the thoracic field requires a wide range of roles, not only morphological diagnosis but also functional analysis and prognosis prediction. There is a greater potential for AI to contribute to thoracic imaging. In addition, the development and validation of further AI technologies may be possible with new imaging techniques such as photon-counting CT [112, 113]. An AI chatbot based on a generative pretrained transformer model (ChatGPT), created by Open AI in 2018, can provide answers across a broad range of diverse topics. While the occurrence of generating inaccurate responses is reduced in a new version GPT-4, it still hampers the practicality of utilizing it in medical education and practice currently [114, 115]. In the future, the combination of GPT-4 and an AI-based CAD system may possibly provide structure reports, which may be useful in both clinical and research support. Furthermore, unlike the traditional AI, Hippocratic AI is receiving a lot of attention as one of the large language models with expertise in tasks related to medical information, such as patient diagnosis, treatment, and answering medical questions. Hippocratic AI is designed to protect patient data and take security measures to ensure proper handling of patients’ personal information, which might provide accurate information and up-to-date medical knowledge for valuable support to healthcare professionals and patients [116]. AI is expected to enable more accurate medical care and provide better personalized medical services to patients. Last of all, it is crucial to remember that AI functions as a tool to assist physicians, and the ultimate decision should be made by the physician while comprehending the limitations of AI.
